# Enhanced Antibacterial Activity of Lactoperoxidase–Thiocyanate–Hydrogen Peroxide System in Reduced-Lactose Milk Whey

**DOI:** 10.1155/2019/8013402

**Published:** 2019-04-23

**Authors:** Ahmad Ni'matullah Al-Baarri, Novia Tri Damayanti, Anang Mohamad Legowo, İsmail Hakkı Tekiner, Shigeru Hayakawa

**Affiliations:** ^1^Food Technology Department, Faculty of Animal and Agricultural Sciences, Diponegoro University, Indonesia; ^2^Animal Sciences Department, Faculty of Animal and Agricultural Sciences, Diponegoro University, Indonesia; ^3^Gastronomy Department, School of Applied Sciences, Istanbul Gelişim University, Turkey; ^4^Applied Biological Sciences, Faculty of Agriculture, Kagawa University, Japan

## Abstract

The product of the lactoperoxidase system (LPOS) has been developed as a preservative agent to inhibit foodborne bacteria, but its action was, heretofore, limited to several original compounds in milk. This research was conducted to analyze the application of the lactoperoxidase system against* Escherichia coli* in fresh bovine milk and its derivative products to determine the strength of antibacterial activity. Lactoperoxidase was purified from bovine whey using the SP Sepharose Big Beads Column. The enzymatic reaction involving lactoperoxidase, thiocyanate, and hydrogen peroxide was used to generate the antibacterial agent from LPOS. This solution was then added to milk, skimmed milk, untreated whey, reduced-LPO whey, reduced-lactose whey, and high-lactose solution containing* E. coli* at an initial count of 6.0 log CFU/mL. LPOS showed the greatest reduction of bacteria (1.68 ± 0.1 log CFU/mL) in the reduced-lactose whey among the products tested. This result may lead to a method for enhancement of the antimicrobial activity of LPOS in milk and derived products.

## 1. Introduction

Lactoperoxidase (LPO) was developed to inhibit the growth of foodborne pathogens in various foods and thus improve their shelf life [1, 2]. Lactoperoxidase derived from bovine milk has been shown to generate beneficial effects as a bactericidal and bacteriostatic agent [1, 3]. The lactoperoxidase system consists of three primary components: lactoperoxidase enzyme, thiocyanate, and hydrogen peroxide. This system generates hypothiocyanite, an active compound against Gram-positive and Gram-negative bacteria, including* Escherichia coli* [4, 5]. The lactoperoxidase system (LPO system) has attracted the attention of scientists as a natural biopreservative with generally recognized as safe (GRAS) status [6]. Hypothiocyanite, a product of LPOS, has been recognized as a safe antibacterial agent without negative effects on human health [7, 8].

Biopreservation using the LPO system could offer an additional hurdle to improve the shelf life of various food products such as fruit [9], chicken meat [10], duck meat [11], cheese [12], and local food products such as dangke [2, 13]. However, slight inhibition of pathogenic bacteria also appeared in fresh milk. Other researchers reported the slight reduction of below 1 log CFU/ml in fresh milk treated with the lactoperoxidase system [14]. It was understood that lactoperoxidase antimicrobial activity might be enhanced using lysozyme [2], beta carotene [15, 16], ectoine [17], alpha tocopherol [18], and chitosan [19], but it was inhibited by several compounds such as hydrogen peroxide and thiocyanate in excess amounts [20–22] and indigenous milk compounds such as casein [23] and saccharides [24]. It was then presumed that the removal of casein and lactose from the milk enabled the use of lactoperoxidase to reduce the population of bacteria in fresh milk.

It was reported that lactose reduces LPO activity by 38% because the sugar molecules interact with the heme cavity of the LPO [24, 25]. The association of sugar molecules with the heme cavity physically blocked the substrate-binding site, thereby resulting in the prevention of the interaction of substrate with the heme iron [21]. This research aims to use LPOS to reduce pathogenic bacteria in milk and its derived products after removal of lactose and casein from milk. This research will provide beneficial information to apply LPOS in milk and derived products.

## 2. Materials and Methods

### 2.1. Materials

SP Sepharose™ Big Beads (Lot No. 10081054) was purchased from GE Healthcare Bio-Sciences AB, Sweden. Microbial rennet was purchased from Prodinvest Group, Russia. Deoxycholate hydrogen sulfide lactose agar (DHL) (Lot No. 395-00461) was obtained from Shinnihonseiyaku Co., Ltd., Japan. ABTS was purchased from Wako Pure Chemical Industry, Japan. Bovine milk was freshly obtained from the experimental farm at the Faculty of Animal and Agricultural Science, Diponegoro University, Semarang, Indonesia. Culture stock of* Escherichia coli *FNCC 0009 was purchased from the Faculty of Agricultural Technology, Gadjah Mada University, Yogyakarta, Indonesia. A spectrophotometer (Mini UV-1240, Shimadzu, Japan) was used for the Bradford protein analysis and enzyme activity. Sterile syringe filters (Lot No. SF2030813) were purchased from Axiva Sichem Biotech Delhi, India. All chemicals used in this study were of analytical grade.

### 2.2. Preparation of Whey, Reduced-Lactose Whey, and High-Lactose Solution

Whey was obtained using fresh bovine milk that was treated with 0.02% (w/v) rennet. Through these treatments, 1 L of fresh bovine milk was converted into 800 mL of whey. Casein was removed using a sterile filter cloth; lactose removal of whey was carried out by dialysis. Untreated whey was dialyzed to produce reduced-lactose whey, and the solution eluted from the dialysis membrane was collected as high-lactose solution.

### 2.3. Purification of LPO from Whey

The procedure for immobilization of LPO from whey was conducted according to the method of previous researchers [25], with minor modifications. SP Sepharose™ Big Beads (SPBB) was used as the matrix for LPO purification from bovine whey. Whey was applied on a glass column (2 x 17 cm) filled with 17 g of SPBB. Preparation of SPBB was initiated by washing with 300 mL pure water and 300 mL of 0.1 mM phosphate buffer (PB) of pH 6.8 containing 1 M NaCl to remove unnecessary compounds. After the whey was applied to the column, the resin was washed with 100 mL 0.4 mM NaCl in 0.1 mM phosphate buffer of pH 7.0 using a fraction collector (10 mL per tube). The purity of the derived LPO was checked by Sodium Dodecyl Sulfate-Polyacrylamide Gel Electrophoresis (SDS-PAGE), using the method of a previous researcher [26]. The protein solution was filtered through a 0.22 *μ*m syringe filter unit. The purified LPO was stored at –20°C. The LPO purification was done for multiple times until the band of LPO showed a clear image using the SDS-PAGE analysis.

### 2.4. Determination of Protein Concentration

Protein content was analyzed using the Coomassie Brilliant Blue reagent [27]. The protein standard was determined using bovine serum albumin.

### 2.5. Inoculum Preparation

The inoculum was prepared following the method of Lang [28] with minor modifications. Before each experiment, stock cultures of* E. coli *FNCC 0009 were streaked onto Nutrient Broth. Cultures were incubated at 39°C for 24 h.

### 2.6. Determination of LPO Activity

LPO activity was assayed using the method of Al-Baarri [24]. A 450 *μ*l aliquot of 1.0 mM ABTS in 10 mM acetate buffer (pH 4.4) and 450 *μ*l 0.55 mM H_2_O_2_ in pure water were poured into the cuvette. Immediately, 50 *μ*l of LPO was added to the cuvette. The increase in absorbance at 412 nm was measured for 1 minute. One unit of LPO enzymatic activity was expressed as the amount of enzyme needed to oxidize 1 *μ*mol ABTS min^−1^. The molar extinction coefficient of ABTS at 412 nm was 32,400 min^−1^ cm^−1^.

### 2.7. Determination of Antibacterial Activity

Antibacterial activity was measured using the method previously described by Touch [10] with modifications. The LPO system, composed of 3.0 U/ml LPO, 0.9 mM KSCN, and 0.9 mM H_2_O_2_, was incubated for 1 hour at room temperature to generate the antibacterial compound. The LPOS solution was then added to the milk and its derivative products, which were inoculated with* E. coli* at approximately 10^7^CFU/mL. Each mixture was incubated in a water bath shaker at 30°C. Controls with 0.1 mM PB of pH 7.0 instead of the milk were subjected to the same treatment as the samples. Serial dilutions in sterilized pure water were prepared to obtain countable numbers of bacteria. Counts were obtained by spreading 100 *μ*L of each mixture onto triplicate plates of DHL. The plates were incubated at 37°C for 24 h. Colony forming units (CFU) were enumerated in plates containing 30–300 colonies, and cell concentration was expressed as log CFU/mL.

### 2.8. Determination of Lactose Content in Whey

Lactose content in whey was determined by using a refractometer. The ability of the refractometer to provide accurate measurements was indicated by how closely the test results matched those obtained with the MilkoScan. This method was adapted from Chigerwe [29]. Whey obtained by the previously mentioned method of whey purification was analyzed by means of the MilkoScan 203 and refractometer, resulting in a mean bias of 94 ± 1.92%. Lactose concentrations were determined by comparing the value obtained by the refractometer with a standard curve generated with lactose. The regression equation with R^2^ = 0.97 was used to determine lactose concentration.

### 2.9. Data Analysis

The analyses for antimicrobial activity and lactose content were carried out in triplicate from 3 independent experiments; then they were analyzed using descriptive analysis to explain their changes. Data are showed as means ± standard error of the mean. Statistical significance was calculated using the GraphPad Prism statistical software (San Diego, USA). The ANOVA analysis was used to decide the significance at* P* values of less than 0.05.

## 3. Result and Discussion

### 3.1. Purification of LPO and Characteristics of the Purified Protein

Lactoperoxidase is known as an antimicrobial agent in milk, saliva, and tears because of its inhibitory action on bacteria through the oxidation reaction involving thiocyanate and hydrogen peroxide [30, 31]. LPO is a glycoprotein consisting of a single polypeptide chain with a molecular weight of 78 kDa (Golhefors and Marklundi, 1975; Jacob et al., 2000). The purification process of LPO from bovine whey was conducted at 10°C to provide optimum binding of LPO to the SP Sepharose matrix [30]. Therefore, this research used SP Sepharose to bind LPO in whey.

**Figure 1:**
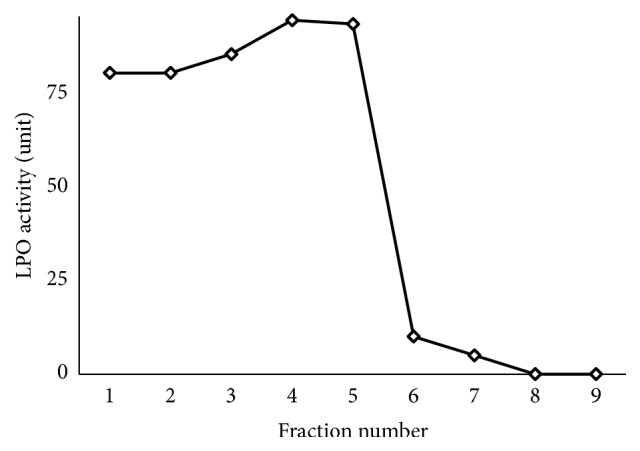
LPO activity in nine fractions obtained from the elution of 0.4 mM NaCl in 0.1 mM phosphate buffer of pH 7.0 through column containing Sepharose™ Big Beads.

**Figure 2:**
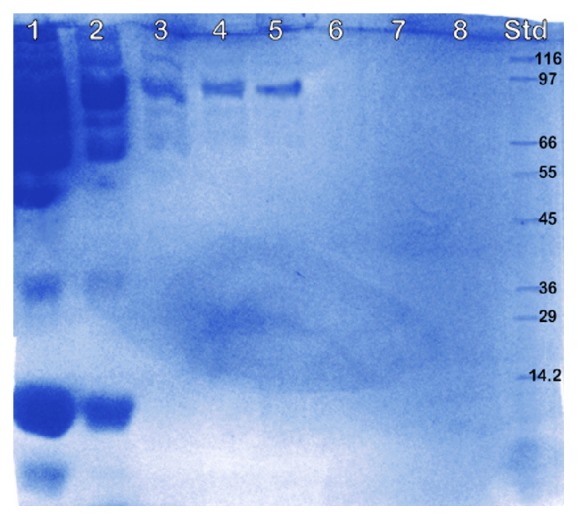
SDS-PAGE profile of nine fractions that were eluted from column packing Sepharose™ Big Beads. Lanes 1 to 8 were the samples from fractions 1 to 8.

A high peak of LPO activity was detected from fraction numbers 1–5, with values in the range of 80–93 units (Figure 1). No significant LPO activity was detected in fractions 6–9. Each fraction was then applied to SDS-PAGE to determine its purity. As a result, several bands were detected in fractions 1–3 (Figure 2). However, fractions 4 and 5 showed a single band with minor other proteins, indicating that purity of LPO was high in these fractions. Therefore, fractions 4 and 5 were mixed and their activity was calculated, obtaining 94 and 93 U/ml, respectively. Since previous application of LPO for reducing* S. enteritidis *only required 4.5 U/ml [16], the lactoperoxidase obtained by this purified LPO sufficiently fulfills the need for LPO application in the next set of experiments. Prior to enzyme collection in a 1.5 ml tube, the mixed fraction was sterilized using a 0.22 *μ*m syringe filter, and then the enzyme was stored at -20°C.

### 3.2. Antibacterial Activity of the LPO System from Bovine Milk

**Figure 3:**
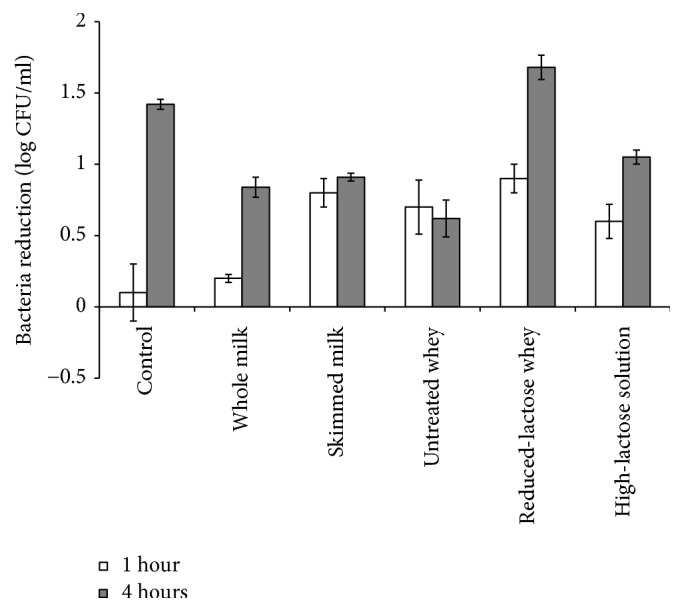
Antibacterial effect of LPOS against* E.coli* in milk and derivative products. This number was calculated from the initial population of 7.0±0.10 log CFU/ml. The solution containing* E. coli *and LPOS was incubated for 1 and 4 hours in 30°C. Values are means ± SE (n = 3).

This research used 7.0 ± 0.10 log CFU/ml of* E. coli* as the initial population. The incubation times were set to 1 and 4 hours at 30°C (Figure 3). It can be seen that LPOS remarkably reduced the population of* E. coli* in PB from the initial count to 5.58 ± 0.10 log CFU/mL, indicating a reduction of 1.42 ± 0.03 log CFU/mL after 4 hours of incubation. These findings imply that inhibitory effects on the antibacterial activity of LPO tend to increase with the higher duration of incubation. However, statistical analysis showed that there were no significant differences (*P* <0.05) in the antibacterial activity among treatments. This might be due to the high population of initial bacteria that was used in this research. The incubation time plays a remarkable role in bacterial reduction that could be seen by the increase in the antibacterial activity at 4 h of incubation. As can be seen in control, antibacterial activity showed less than 0.1 log CFU/mL in the sample with a 1-hour incubation and then elevated to 1.42±0.04 CFU/mL at 4 h incubation. Opstal [32] reported a greater reduction of* E. coli* by LPOS (2.2 log CFU/mL) from the initial count of 6.0 CFU/mL during 6 hours of incubation at 20°C. These differences between studies might have been due to differences in the bacterial load and incubation time.

Bacterial reductions in whole milk, skimmed milk, and untreated whey were less than in the control (<1.0 log CFU/mL), possibly due to the presence of casein and lactose in milk and whey. Casein is the abundant component in milk protein that might protect substrate microorganisms from absorption of the antimicrobial component, thus weakening the inhibitory effect on bacteria [23]. It is known that bactericidal effects of OSCN^–^ compounds from LPOS are key to kill bacteria by disrupting sulfhydryl groups (-SH) on proteins from the bacterial cytoplasmic membrane [24], so the interaction between the sulfhydryl group and OSCN^–^ might be hindered, resulting in the weakening of antibacterial action. Inhibition of LPOS action could also occur due to hydrogen peroxide released from bacteria [23].

The lactose content in untreated whey was 1.82 ± 0.20%, and after dialysis, it was reduced to 0.69 ± 0.10% (Table 1). Results from the statistical study showed that the reduction exhibited no significant difference (*P* <0.05), but it showed 62% reduction resulting in the 2.7 times antibacterial activity enhancement of LPO from 0.62 ± 0.20 to 1.68 ± 0.10 that clearly indicated inhibition of antibacterial activity of LPOS by lactose (Figure 3). These results were corroborated by those of previous researchers [10], finding that LPOS was unable to reduce the significant amount of* S. enteritidis* in whole milk. Saccharides including lactose were potent inhibitors of lactoperoxidase activity and showed kinetic inhibition of 3.20 ± 0.52 [24]. Therefore, the reduction of the lactose amount in milk might increase the action of LPOS against the growth of bacteria. The inhibition of LPOS by lactose might be due to the weakening of enzymatic activity of LPO, since saccharides are a nonspecific stabilizer of protein that allows for direct interaction between carbohydrate and protein molecules through hydrogen bond formation, resulting in the reduction of enzymatic activity [33]. In addition, as reported by previous researchers [34], the carboxylic group might bind to the side chain of 2-Glu258 to form a strong hydrogen bond resulting in the inability of a natural substrate such as thiocyanate to bind to LPO.

It was described that lactose had performed as an LPO inhibitor; therefore the lactose conversion into another compound was suggested. Previous researchers [35] applied lactose reduction using lactose oxidase to generate an H_2_O_2_ compound resulting in the enhancement of antimicrobial function of LPOS; however the avoidance of lactose binding to the specific site of LPO might be required since lactose may still provide beneficial effect to the nutrient content of a dairy product. However, in order to achieve the practical application in the dairy industry, this research may provide the novelty with clear explanation that the reduction of lactose content is strongly suggested to exhibit the beneficial impact on the shelf life of dairy products.

## 4. Conclusion

This research indicated that LPOS had moderate antibacterial effects on* E. coli *in whole milk, skimmed milk, and whey. Lactose reduction from whey remarkably enhanced bactericidal activity. LPOS can effectively act as an antibacterial reagent in reduced-lactose dairy products.

## Figures and Tables

**Table 1:** Lactose content in untreated whey, reduced-lactose whey, and high-lactose solution.

Materials	Lactose content (%)

Untreated whey	1.82±0.20
Reduced-lactose whey	0.69±0.10
High-lactose solution	2.05±0.30

Values are means ± SE (n = 5).

## Data Availability

The authors state that the data in this article were obtained as naturally as possible with the proper replication. The authors also state that the previously reported “lactoperoxidase–thiocyanate–hydrogen peroxide system” was used to support this study and is available at http://doi.org/10.3923/ijds.2014.116.123 and https://doi.org/10.4315/0362-028X.JFP-10-184. These prior studies are cited at relevant places within the text as references: [13] Rasbawati, A.N. Al-Baarri, A.M. Legowo, V.P. Bintoro, B. Dwiloka, Total bacteria and pH of dangke preserved using natural antimicrobial lactoferrin and lactoperoxidase from bovine whey, International Journal of Dairy Science, 9 pp. 116–123, 2014; [24] A.N. Al-Baarri, M. Hayashi, M. Ogawa, S. Hayakawa, Effects of mono- and di-saccharides on the antimicrobial activity of bovine lactoperoxidase system, Journal of Food Protection, 74 pp. 134–139, 2011.
